# Impact of Different e-Cigarette Generation and Models on Cognitive Performances, Craving and Gesture: A Randomized Cross-Over Trial (CogEcig)

**DOI:** 10.3389/fpsyg.2017.00127

**Published:** 2017-03-09

**Authors:** Pasquale Caponnetto, Marilena Maglia, Maria Concetta Cannella, Lucio Inguscio, Mariachiara Buonocore, Claudio Scoglio, Riccardo Polosa, Valeria Vinci

**Affiliations:** ^1^Centro per la Prevenzione e Cura del Tabagismo, Azienda Ospedaliero-Universitaria “Policlinico-V. Emanuele,” Università di CataniaCatania, Italy; ^2^Institute of Internal Medicine, G. Rodolico Hospital, Azienda Ospedaliero-Universitaria “Policlinico-V. Emanuele,” Università di CataniaCatania, Italy; ^3^Institute for Social Marketing, University of StirlingStirling, UK; ^4^Psychology Service, ASP CataniaCatania, Italy; ^5^Department of Psychology, University of Rome La SapienzaRome, Italy; ^6^Department of Clinical Neurosciences, San Raffaele Scientific InstituteMilan, Italy

**Keywords:** smoking cessation, smoking reduction, cognition, adverse events, electronic cigarettes, electronic nicotine delivery devices, cigarette substitutes

## Abstract

**Introduction:** Most electronic-cigarettes (e-cigarette) are designed to look like traditional cigarettes and simulate the visual, sensory, and behavioral aspects of smoking traditional cigarettes. This research aimed to explore whether different e-cigarette models and smokers' usual classic cigarettes can impact on cognitive performances, craving and gesture.

**Methods:** The study is randomized cross-over trial designed to compare cognitive performances, craving, and gesture in subjects who used first generation electronic cigarettes, second generation electronic cigarettes with their usual cigarettes. (Trial registration: ClinicalTrials.gov number NCT01735487).

**Results:** Cognitive performance was not affected by “group condition.” *Within-group* repeated measures analyses showed a significant time effect, indicating an increase of participants' current craving measure in group “usual classic cigarettes (group C),” “disposable cigalike electronic cigarette loaded with cartridges with 24 mg nicotine (group H), second generation electronic cigarette, personal vaporizer model Ego C, loaded with liquid nicotine 24 mg (group E). Measures of gesture not differ over the course of the experiment for all the products under investigation

**Conclusion:** All cognitive measures attention, executive function and working memory are not influenced by the different e-cigarette and gender showing that in general electronics cigarettes could become a strong support also from a cognitive point of view for those who decide to quit smoking. It seems that not only craving and other smoke withdrawal symptoms but also cognitive performance is not only linked to the presence of nicotine; this suggests that the reasons behind the dependence and the related difficulty to quit smoking needs to be looked into also other factors like the gesture.

Clinical Trial Registration: www.ClinicalTrials.gov, identifier NCT01735487.

## Introduction

Cigarette smoking is the single most important cause of avoidable premature mortality in the world and quitting is known to rapidly reduce risk of serious diseases such as lung cancer, cardiovascular disease, strokes, chronic lung disease and other cancers^1^^,^^2^. The World Health Organization (WHO) Framework Convention on Tobacco Control (FCTC) advises that the key to reducing the health burden of tobacco is to encourage abstinence among smokers^3^.

Electronic cigarettes (E-cigarettes) are battery-operated devices designed to vaporize a liquid solution of propylene glycol and vegetable glycerin in which nicotine or other aromas may be dissolved (Hon, 2005). Puffing activates a battery-operated heating element in the atomizer and the liquid in the cartridge is vaporized as a plume of a dense mist and inhaled. Because e-cigarettes do not burn tobacco, these products may be considered as a lower risk substitute for factory-made cigarettes (Caponnetto et al., 2012). Most E-cigarettes are designed to look like traditional cigarettes and simulate the visual, sensory, and behavioral aspects of smoking traditional cigarettes (Caponnetto et al., 2013a). Recent internet survey on the satisfaction of E-cigarette use has reported that the device helped in smoking abstinence and improved smoking-related symptoms (Etter, 2010; Dawkins et al., 2013a; Goniewicz et al., 2013). Moreover, several studies has reported that the electronic cigarette helped in smoking cessation and smoking reduction (Polosa et al., 2011, 2013; Caponnetto et al., 2013b). These notions, indicate that the e-cigarettes may be an effective and safe cigarette substitute, and therefore merits further evaluation for this purpose. Moreover, several studies support the hypothesis that classic and electronic cigarette aids cognitive performances (McEwen et al., 2008; Heishman et al., 2010; Dawkins et al., 2012, 2013b). Studying e-cigarettes' impact on cognitive functioning could help understanding the mechanisms underlying smoking cessation. Smoking cessation requires also a great deal of restraint over an extended period of time and it may be fruitful to consider the quitting process as a test of the smoker's ability to delay the short-term gratification from smoking a classic cigarette in favor of the long-term health benefits associated with cessation. Some researchers studied the gratification as a variable to quit smoking (Mueller et al., 2009; Wilson et al., 2015).

This research aimed to explore whether different e-cigarette models can impact on craving, gesture and cognitive performances. We used three neurocognitive tests (WCST, CPT-AX, N-BACK) to compare cognitive performances in subjects who used first generation rechargeable cigalike, e-cigarettes, loaded with cartridges 24 mg nicotine, tobacco aroma, second generation, disposable cigalike electronic cigarette loaded with cartridges with 24 mg nicotine, tobacco aroma, second generation disposable cigalike electronic cigarette loaded with cartridges with 0 mg nicotine, mint aroma, second generation electronic, personal vaporizer, model Ego C (tank cartomizer), loaded with liquid nicotine 24 mg, tobacco aroma, with their usual cigarettes.

Our starting hypothesis was that the high nicotinic release of classic cigarettes compared to electronics could translate into better cognitive performance, better reduction of craving and greater gestural satisfaction.

## Methods

### Participants

Thirty four regular smokers were recruited, during the period May 2015–September 2015, in Catania, Italy. No financial incentive was offered for participation. The first consecutive 34 eligible smokers were included in the study conducted at Centro per la Prevenzione e Cura del Tabagismo–(CPCT; Universita‘ di Catania, Italy). Eligible participants were invited to visit the study center, complete a questionnaire asking about demographic and smoking characteristics and undergo screening/baseline visit (medical history, blood pressure, heart rate, exhaled carbon monoxide and assessment of physical and behavioral cigarette dependence).

Inclusion criteria were: (a) smoke ≥15 factory made cigarettes per day (cig/day), for at least the past 10 years, (b) age 18–70 years, (c) in good general health; and (d) committed to follow the trial procedures.

Exclusion criteria were: (a) symptomatic cardiovascular disease; (b) symptomatic respiratory disease; (c) regular psychotropic medication use; (d) current or past history of alcohol abuse; (e) use of smokeless tobacco or nicotine replacement therapy, and (f) pregnancy or breastfeeding.

The study was approved by the local institutional ethics committee. (Clinical trial registration http://clinicaltrials.gov/show/NCT01735487).

### Study design and screening/baseline measures

The study is randomized cross-over trial designed to compare cognitive performances, craving and gesture in subjects who used first generation rechargeable cigalike, e-cigarette, loaded with cartridges 24 mg nicotine, tobacco aroma, second generation, disposable cigalike electronic cigarette loaded with cartridges with 24 mg nicotine, tobacco aroma, second generation disposable cigalike electronic cigarette loaded with cartridges with 0 mg nicotine, mint aroma, second generation electronic, personal vaporizer, model Ego C (tank cartomizer), loaded with liquid nicotine 24 mg, tobacco aroma, with their usual cigarettes.

Eligible participants were invited to write informed consent, complete a questionnaire asking about demographic and smoking history/characteristics, complete Fagerström Test for Cigarette Dependence (FTCD) (Fagerstrom and Schneider, 1989), Glover-Nilsson Smoking Behavioral Questionnaire (GN-SBQ) (Glover et al., 2005), and undergo screening (medical history, blood pressure, heart rate). The Fagerström Test for Cigarette Dependence is a standard instrument for assessing the intensity of physical addiction to nicotine. The test was designed to provide an ordinal measure of nicotine dependence related to cigarette smoking. It contains six items that evaluate the quantity of cigarette consumption, the compulsion to use, and dependence (Fagerstrom and Schneider, 1989). The Glover-Nilsson Smoking Behavior Questionnaire (GN-SBQ) is a self-report measure of behavioral dependence based on behaviors that may surround smoking or thoughts about smoking (Glover et al., 2005). Additionally, levels of carbon monoxide in exhaled breath (eCO) were measured using a portable device (Micro CO, Micro Medical Ltd, UK).

“Cigarette craving” and “smoking gesture” were measured by asking, “Right now, how much do you want a cigarette?” “Right now, how much do you want a cigarette in your hand or in your mouth?” We asked participants to indicate their perception of all items by circling a visual analog scale number between 0 and 10, where 0 = “not at all” and 10 = “extremely.”

### Procedures

At each study visit participants were requested to abstain from smoking and alcohol from 20:00 on the night before each study day and from food and caffeine for at least 1 h before the session. On arrival at the study center, carbon monoxide (CO) was measured in participants' expired breath using a portable device (Micro CO, Micro Medical Ltd, UK). If CO was ≤10 parts per million (ppm), the assigned study treatment was allocated; however, if CO was >10 ppm or they reported smoking in the previous 12 h, participants were rescheduled wherever possible to a subsequent session. On the first study day, participants were randomized to use one of five different products: first generation rechargeable cigalike, e-cigarettes, loaded with cartridges 24 mg nicotine, tobacco aroma; second generation, disposable cigalike electronic cigarette loaded with cartridges with 24 mg nicotine, tobacco aroma; second generation disposable cigalike electronic cigarette loaded with cartridges with 0 mg nicotine, mint aroma; second generation electronic, personal vaporizer, model Ego C (tank cartomizer), loaded with liquid nicotine 24 mg, tobacco aroma; with their usual classic cigarettes.

Allocation was performed using a random sequence of five codes, each corresponding to one product, prepared in advance by the study statistician using the Latin-square method to control for time effects.

Time 1 (T1) participants sat at desks in a room where they completed ratings of craving, gesture 3 min before using their allocated product. They took their first electronic or classic cigarette at 08:00 a.m. Participants randomized to a day using the e-Cigarette or their usual cigarette will be asked to puff the study product for 3 min to take 15 puffs (Puffing Acute Phase). After the first hour, they will leave the study center and will use the study product as required for a further at least 10 h.

Ratings were made at 3 (T2), 5 (T3), 7 (T4), 17 (T5), and 32 (T6) min counting from the first 15 puff on each product.

### Cognitive assessment

The following neuro-cognitive tests were completed, 5 min counting from the first 15 puff on each product, at the each visits: Continuous Performance Test—AX version (CPT-AX) (Rosvold et al., 1956), Wisconsin card sorting test (WCST) (Psychological Assessment Resources, 2003), Working memory test (N-BACK) (Kirchner, 1958).

CPT-AX is the most popular clinical measures of sustained attention and vigilance. The basic paradigm for CPTs involves selective attention or vigilance in response to an infrequently occurring stimulus. It is characterized by rapid presentation of continuously changing stimuli with a designated “X” stimulus (Rosvold et al., 1956). The Wisconsin Card Sorting Test (WCST), originally developed to assess abstract reasoning ability and the ability to shift cognitive strategies in response to changing environ-mental contingencies, is also considered a measure of the executive functions. WCST requires strategic planning, organized searching, utilizing environmental feedback to shift cognitive sets, directing behavior toward achieving a goal and modulating impulsive responding (Psychological Assessment Resources, 2003). The N-Back Test was developed as a way to measure working memory. In this test, the subject is given a sequence of stimuli, shown in order (Kirchner, 1958).

Following study completion, participants were invited to attend a smoking cessation treatment at Centro per la Prevenzione e Cura del Tabagismo (CPCT), AOU “Policlinico Vittorio Emanuele” University of Catania. Twenty one participants followed a tailored smoking cessation treatment.

### Products tested

(a) First generation rechargeable cigalike, e-cigarettes, loaded with cartridges 24 mg nicotine **(model “401”)**.

The e-Cigarette “Categoria” model “401” was been supplied by the manufacturer, Arbi Group Srl (Milano, Italy). It is a three-piece model that closely resembles a tobacco cigarette. Its heating element in the atomizer is activated by a rechargeable 3.7 V–90 mAh lithium-ion battery. A fully charged battery can last up to the equivalent of 50–70 puffs. Disposable cartridges used in this study looked like tobacco cigarette's filters containing an absorbent material saturated with a liquid solution of propylene glycol and vegetable glycerin in which nicotine or an aroma was dissolved. Disposable cartridges had to fit securely onto the heating element of the atomizer in order to produce a consistent vapor. One types of cartridges was provided for this study day; “Original” 24 mg nicotine. Detailed toxicology and nicotine content analyses of these cartridges had been carried in a laboratory certified by the Italian Institute of Health and can be found at: http://www.categoriacigarette.com/. The cartridge labeled “Original 24 mg” contains liquid comprising 1.4% water, 2.37% nicotine, 75.6% propylene glycol, ethanol 0.16, glycerine 19.7%, pyrazine, trimentyl 0.10%, 2,3-dimethylpyrazine 0.13%, myosmine 0.15%.

(b) Second generation, *disposable cigalike* electronic cigarette loaded with cartridges with 24 mg nicotine, **(model 501 “ONE original”)**.

This is a single use electronic cigarette. Compared to “Categoria” Electronic Cigarette (model “501”), the model ONE high original has a new filter technology that comprises an integrated atomizer and a new long life battery, which guarantee high performance. Externally, these electronic cigarettes resemble conventional cigarettes; but internally, they contain a lithium battery, a heater unit, an integrated circuit, and a wick surrounded by a cotton wad containing 0.5 mL of nicotine solution. These electronic cigarettes are neither rechargeable nor refillable; rather, they are disposable. The nicotine solution contains approximately 24 mg of nicotine. Detailed toxicology and nicotine content analyses of these cartridges had been carried in a laboratory certified by the Italian Institute of Health and can be found at: http://www.categoriacigarette.com/it/studi-e-ricerche/analisi/analisi-2013. The cartridge contains liquid comprising 2.2% Nicotine, 21.2% Glycerine, 70.8% Propylene Glicol, <0.1% Ethylene Glicol, 4.5% Water, 0.4% Flavors and Additives, <5% Cadmium l, <5% Lead, <1% Mercury, <5% Chromium.

(c) Second generation *disposable cigalike* electronic cigarette loaded with cartridges with 0 mg nicotine, mint aroma **(model 501 “ONE Mint”)**.

This is a single use electronic cigarette. Compared to **“Categoria” Electronic Cigarette (model “501”)**, the model ONE Mint has a new filter technology that comprises an integrated atomizer and a new long life battery, which guarantee high performance. Externally, these electronic cigarettes resemble conventional cigarettes; but internally, they contain a lithium battery, a heater unit, an integrated circuit, and a wick surrounded by a cotton wad containing 0.5 mL of nicotine solution. These electronic cigarettes are neither rechargeable nor refillable; rather, they are disposable. The nicotine solution no contains nicotine Detailed toxicology and nicotine content analyses of these cartridges had been carried in a laboratory certified by the Italian Institute of Health and can be found at: http://www.categoriacigarette.com/it/studi-e-ricerche/analisi/analisi-2013. The cartridge contains liquid comprising <0.001% Nicotine, 18.8% Glycerine, 72.5% Propylene Glicol, 2.1% Ethylene Glicol, 4.9% Water, 0.78% Flavors and Additives, <5% Cadmium l, <5% Lead, <1% Mercury, <5% Chromium.

(d) Second generation electronic, personal vaporizer, model Ego C (tank cartomizer), loaded with liquid nicotine 24 mg, tobacco aroma.

The e-Cigarette (“Ego”) were supplied by, Fumo digitale (Varese, Italy). The electronic cigarette Ego C (Joyetech), used in the study, consist of the atomizer, the tank cartomizers and the battery. This electronic cigarette is considered—second generation; the battery has higher capacity compared to cigarette-like devices and the atomizer design is different compared to polyfil-containing cartomizers. A 24 mg/ml nicotine-containing liquid was used (Tuscan flavor by Flavouart), which is generally considered high strength.

The E-liquid Tuscan by Flavourart were supplied by Flavourart (Oleggio-NO, Italy). This E-liquid comprising 0.80 g USP Nicotine, 44.82 g USP Glycerine, Propylene Glicol USP 46.7 g, 8.11 g Water, <0.5 g Flavors.

(e) Participants usual classic cigarettes.

### Data analysis

Responses were investigated with analyses of variance for repeated measures with SPSS for Windows, Version 19.1 (IBM Corp Released, 2010). The multivariate solution was used for repeated measures factors with more than two levels. Details of individual analyses are described in the Results.

Data were analyzed using General linear model—ANOVA repeated measures analyses were conducted for each test session between different group conditions: First generation rechargeable cigalike, e-cigarettes, loaded with cartridges 24 mg nicotine, tobacco aroma, named “O”—Original 24 mg nicotine; Second generation, disposable cigalike electronic cigarette loaded with cartridges with 24 mg nicotine, tobacco aroma, named “H”—One High 24 mg nicotine; Second generation disposable cigalike electronic cigarette loaded with cartridges with 0 mg nicotine, mint aroma named “N”—Mint nicotine free; Personal vaporizer, model Ego C (tank cartomizer), loaded with liquid nicotine 24 mg, tobacco aroma named “E”; Usual own daily classic cigarettes labeled “C.”

Data were analyzed to explore between-group differences with respect to the performance in the following tests: Continuous Performance Test—AX version (CPT-AX), Wisconsin Card Sorting Test (WCST), Working Memory test (N-BACK). Also, data were analyzed to explore between-group difference with respect to the dependent variables:

- Craving: in T1,T2,T3,T4,T5,T6. Participants rated their current desire for a cigarette using single item visual analog scale number between 0 and 10, where 0 = “not at all” and 10 = “extremely.”
- Carbon monoxide in exhaled breath (eCO) was measured at T1,T2,T3,T4,T5.
- Gesture: at minute 2 during the 15 puff. Participants rated their gesture satisfaction for the specific product using a single item visual analog scale number between 0 and 10 (“Right now, how much do you want a cigarette in your hand or in your mouth?”), where 0 = “completely unsatisfying,” 10 = “fully satisfying.”

### Baseline assessment measures

Demographic information including age (mean = 34.8, *sd* = 11.4), gender (*M* = 20, *F* = 4) education (middle school diploma = 3; high school diploma = 22; bachelor's degree was collected = 9);

## Results

### Cognitive assessment

Table 1 show the means and SDs for each group in CPT AX test. The CPT AX variable were normally distributed, overall attention assessed by CPT AX performance was not affected by “group condition” (C,H,O,E,N) and there were no interactions between “group condition” and gender.

**Table 1:** **Means and SDs for each group in CPT AX test**.

	**Mean**	**SD**	***P***	***N***
Continuous Performance Test – AX version/Own classic cigarettes	9.56	10.835	NS	34
Continuous Performance Test – AX version/Second generation, disposable cigalike electronic cigarette loaded with cartridges with 24 mg nicotine	10.59	11.319	NS	34
Continuous Performance Test – AX version/First generation rechargeable cigalike, e-cigarettes, loaded with cartridges 24 mg nicotine	10.91	12.657	NS	34
Continuous Performance Test – AX version/Second generation disposable cigalike electronic cigarette loaded with cartridges with 0 mg nicotine, mint aroma	11.29	16.552	NS	34
Continuous Performance Test – AX version/Personal vaporizer, model Ego C (tank cartomizer), loaded with liquid nicotine 24 mg, tobacco aroma	10.91	12.636	NS	34

Table 2 show the means and SDs for each group in WSCT test. The WSCT variable was normally distributed, Overall executive functioning assessed by WSCT performance wasn't affected by “group condition” (C,H,O,N,E) and there were no interactions between group condition and gender.

**Table 2:** **Means and SDs for each group in WSCT test**.

	**Mean**	**SD**	***P***	***N***
Wisconsin card sorting test/Own classic cigarettes	10.91	9.983	NS	34
Wisconsin card sorting test/Second generation, disposable cigalike electronic cigarette loaded with cartridges with 24 mg nicotine	7.18	6.525	NS	34
Wisconsin card sorting test/First generation rechargeable cigalike, e-cigarettes, loaded with cartridges 24 mg nicotine	8.12	11.393	NS	34
Wisconsin card sorting test/Second generation disposable cigalike electronic cigarette loaded with cartridges with 0 mg nicotine, mint aroma	8.06	7.075	NS	34
Wisconsin card sorting test/Personal vaporizer, model Ego C (tank cartomizer), loaded with liquid nicotine 24 mg, tobacco aroma	8.18	8.709	NS	34

Table 3 show the means and SDs in n-back 1 task.

**Table 3:** **n-back-1 performance**.

	**Mean**	**SD**	***P***	***N***
n-back-1/Own classic cigarettes	40.24	24.360	NS	34
n-back-1/Second generation, disposable cigalike electronic cigarette loaded with cartridges with 24 mg nicotine	37.65	29.511	NS	34
n-back-1/First generation rechargeable cigalike, e-cigarettes, loaded with cartridges 24 mg nicotine	37.29	29.864	NS	34
n-back-1/Second generation disposable cigalike electronic cigarette loaded with cartridges with 0 mg nicotine, mint aroma	32.82	32.027	NS	34
n-back-1/Personal vaporizer, model Ego C (tank cartomizer), loaded with liquid nicotine 24 mg, tobacco aroma	38.71	28.228	NS	34

Table 4 show the means and SDs for in n-back 2 task.

**Table 4:** **n-back-2 performance**.

	**Mean**	**SD**	***P***	***N***
n-back-2/Own classic cigarettes	60.24	18.656	NS	34
n-back-2/Second generation, disposable cigalike electronic cigarette loaded with cartridges with 24 mg nicotine	62.03	18.252	NS	34
n-back-2/First generation rechargeable cigalike, e-cigarettes, loaded with cartridges 24 mg nicotine	53.76	21.079	NS	34
n-back-2/Second generation disposable cigalike electronic cigarette loaded with cartridges with 0 mg nicotine, mint aroma	51.29	20.877	NS	34
n-back-2/Personal vaporizer, model Ego C (tank cartomizer), loaded with liquid nicotine 24 mg, tobacco aroma	54.32	21.087	NS	34

The nba/1-nba/2 variables were normally distributed, overall n-back performance was not affected by “group condition” (C,H,O,N,E) and there were no interactions between group condition and gender.

**Figure 1:**
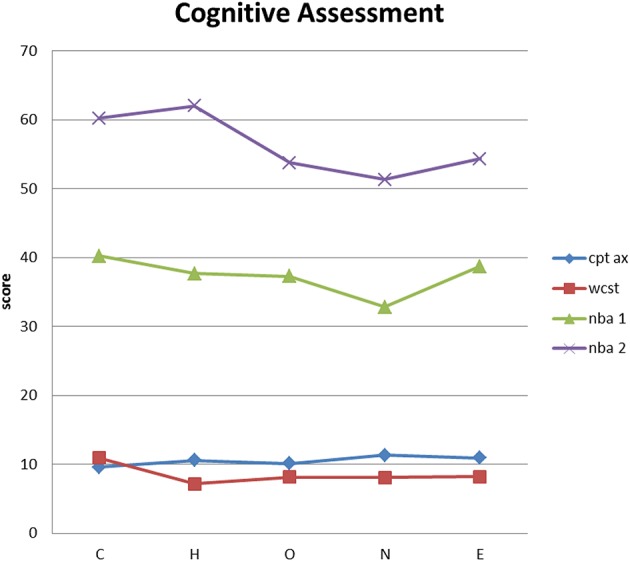
**Means for each group in CPT AX, WCST, N-BACK version 1 and 2, test**.

Figure 1 show the means for each group in CPT AX, WCST, N-BACK version 1 and 2, test.

### eCo dependent measure

*Within-group* repeated measures analyses showed a significant time effect, indicating an increase of participants' current eCo for cigarettes from T1 to T5 in group “C.”

**Figure 2:**
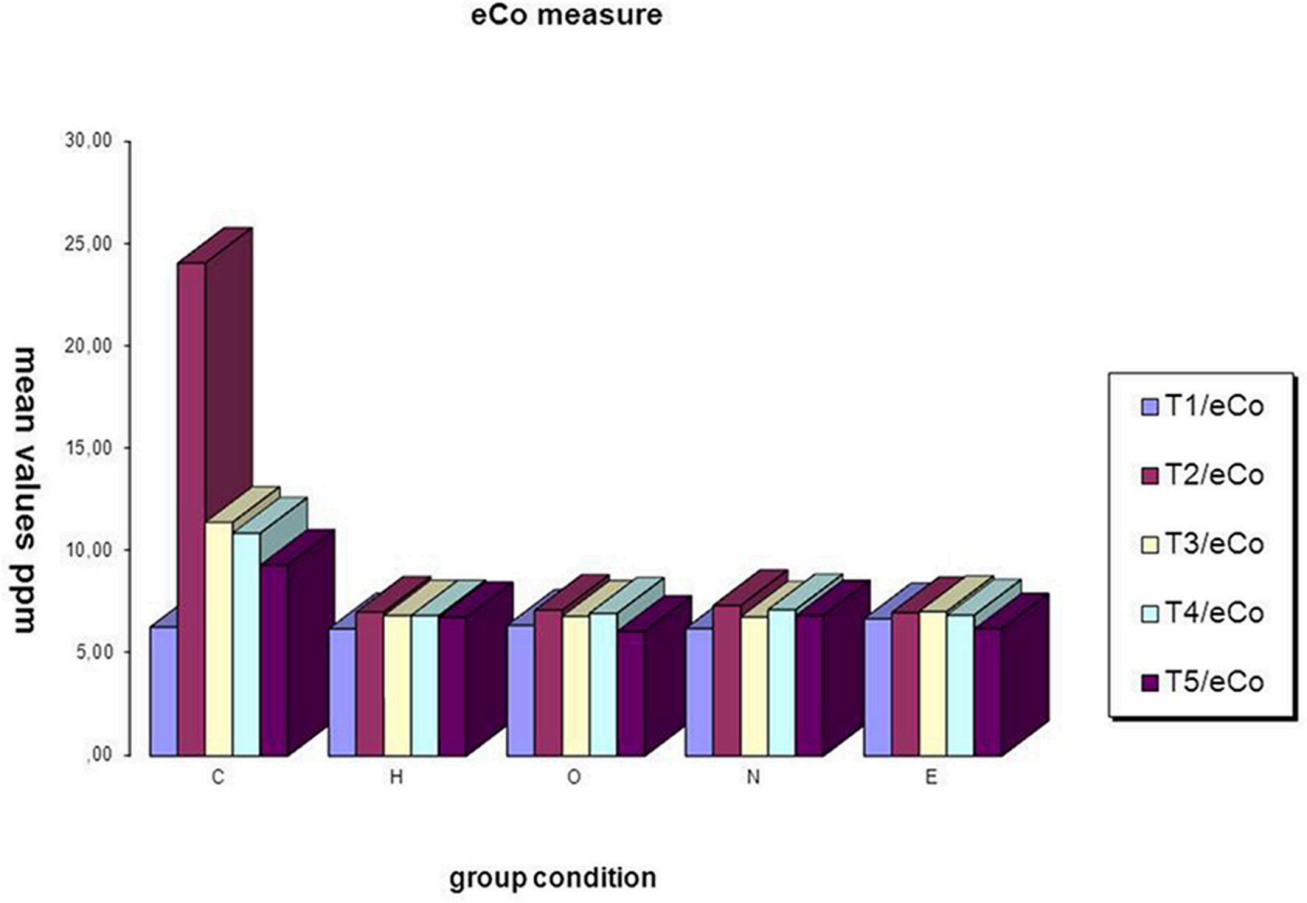
**Means for each group in eCo**.

From T1 to T2 the eCo mean value increases in “C” group [*F*_(1, 33)_ = 25.1 and *p* < 0.001] (Figure 2), but from T2 to T3, from T3 to T4 and form T4 to T5 observation time the mean values decrease significantly in C,O,E group (Table 5).

**Table 5:** **eCo dependent measure**.

**Source**	**Group**	**Comparison**	***F***	**Sig**.
eCO	C (Own classic cigarettes)	T1 (*m* = 6.24, *sd* = 0.5) vs. T5 (*m* = 9.3, *sd* = 0.9)	25.178	0.000
		T2 (*m* = 24.05, *sd* = 2.9) vs. T5 (*m* = 9.3, *sd* = 0.9)	28.398	0.000
		T3 (*m* = 11.4, *sd* = 1.1) vs. T5 (*m* = 9.3, *sd* = 0.9)	9.289	0.005
		T4 (*m* = 10.8, *sd* = 1.04) vs. T5 (*m* = 9.3, sd = 0.9)	18.625	0.000
	O (First generation rechargeable cigalike, e-cigarettes)	T1 (*m* = 6.38, *sd* = 0.50) vs. T5 (*m* = 6.08, *sd* = 0.59)	1.197	0.02
		T2 (*m* = 7.1, *sd* = 0.64) vs. T5 (*m* = 6.08, *sd* = 0.59)	30.509	0.000
		T3 (*m* = 6.8, *sd* = 0.61) vs. T5 (*m* = 6.08, *sd* = 0.59)	13.596	0.001
		T4 (*m* = 6.9, *sd* = 0.59) vs. T5 (*m* = 6.08, *sd* = 0.59)	23.276	0.000
	E (Personal vaporizer, model Ego C)	T1 (*m* = 6.07, *sd* = 0.58) vs. T5 (*m* = 6.2, *sd* = 0.55)	3.573	0.048
		T2 (*m* = 7.00, *sd* = 0.60) vs. T5 (*m* = 6.2, *sd* = 0.55)	10.793	0.002
		T3 (*m* = 7.05, *sd* = 0.65) vs. T5 (*m* = 6.2, *sd* = 0.55)	7.144	0.012
		T4 (*m* = 6.8, *sd* = 0.56) vs. T5 (*m* = 6.2, *sd* = 0.55)	5.061	0.031

### Craving dependent measure

*Within-group* repeated measures analyses showed a significant time effect, indicating an increase of participants' current Craving measure in group “C,” “H” “E.” C group [*F*_(5, 29)_ = 18.3 and *p* < 0.001]. H group [*F*_(5, 29)_ = 3.9 and *p* < 0.001]. E group [*F*_(5, 29)_ = 4.19 and *p* < 0.001] (Table 6).

**Table 6:** **Craving dependent measure**.

**Source**	**Group**	**Comparison**	***F***	**Sig**.
Craving	C (Own classic cigarettes)	T1 (*m* = 36, *sd* = 0.14) vs. T6 (*m* = 3.7, *sd* = 0.5)	33.030	0.000
		T2 (*m* = 0.70, *sd* = 0.22) vs. T6 (*m* = 3.7, *sd* = 0.5)	25.588	0.000
		T3 (*m* = 0.73, *sd* = 0.21) vs. T6 (*m* = 3.7, *sd* = 0.5)	27.448	0.000
		T4 (*m* = 2.04, *sd* = 0.42) vs. T6 (*m* = 3.7, *sd* = 0.5)	11.310	0.002
		T5 (*m* = 2.24, *sd* = 0.40) vs. T6 (*m* = 3.7, *sd* = 0.5)	10.941	0.003
	H (Second generation, disposable cigalike electronic cigarette loaded with cartridges with 24 mg nicotine)	T1 (*m* = 2.6, *sd* = 0.60) vs. T6 (*m* = 4.8, *sd* = 0.65)	11.295	0.002
		T2 (*m* = 2.7, *sd* = 0.53) vs. T6 (*m* = 4.8, *sd* = 0.65)	14.946	0.001
		T3 (*m* = 2.8, *sd* = 0.52) vs. T6 (*m* = 94.8, *sd* = 0.65)	16.427	0.000
		T4 (*m* = 3.2, *sd* = 0.55) vs. T6 (*m* = 4.8, *sd* = 0.65)	16.976	0.000
		T5 (*m* = 3.9, *sd* = 0.54) vs. T6 (*m* = 4.8, *sd* = 0.65)	5.862	0.022
	E (Personal vaporizer, model Ego C)	T1 (*m* = 2.5, *sd* = 0.6) vs. T6 (*m* = 4.4, *sd* = 0.6)	10.585	0.003
		T2 (*m* = 2.6, *sd* = 0.5) vs. T6 (*m* = 4.4, *sd* = 0.6)	10.889	0.003
		T3 (*m* = 6.24, *sd* = 0.6) vs. T6 (*m* = 4.4, *sd* = 0.6)	4.279	0.048

In N and O group condition, we don't have a Craving effect differences.

In C group, craving measure increase over the course of the experiment from T1 to T6 time ratings; also, in H group, craving measure increase over the course of the experiment from T1 to T6 time ratings.

**Figure 3:**
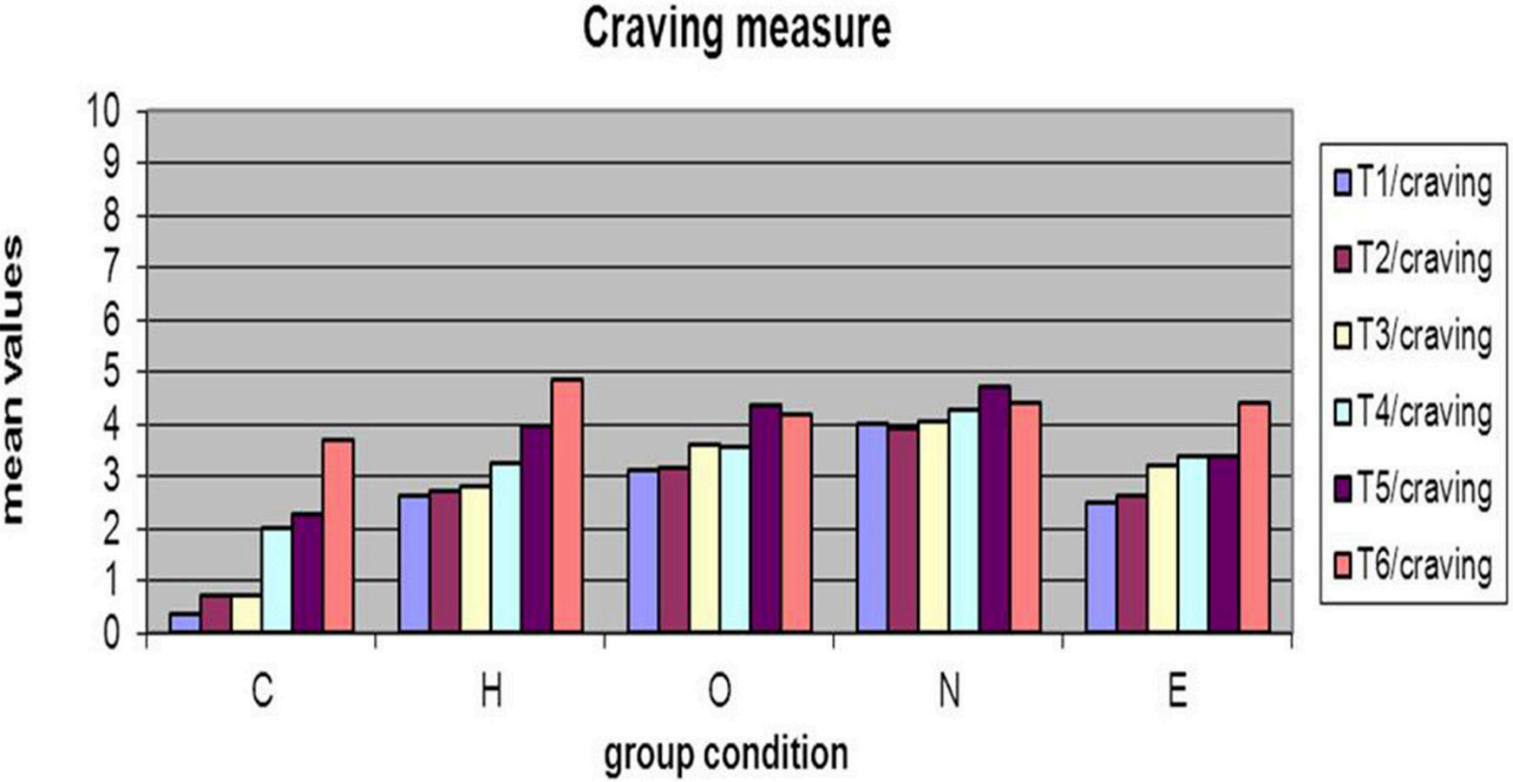
**Means for each group in Craving measures**.

In E group, craving measure increase over the course of the experiment form T1 to T3 time ratings (see Figure 3).

### Gesture measure

Measures of Gesture not differ over the course of the experiment for all study product.

## Discussion

The aim of the study was to compare different cognitive performance in subjects that have used different electronic cigarettes. In literature, there are discordant results about the nicotine effect on cognitive performance. In detail, only one study has investigated the electronic-cigarette effect on cognition. Dawkins et al. (2012) enrolled 85 smoking patients, that have been randomly divided into three groups, each under one of the following conditions: (i) 18 mg nicotine e-cigarette (nicotine), (ii) 0 mg e-cigarette (placebo) (iii) just hold the e-cigarette conditions. It has been noticed that in the subjects belonging to the first group there is been a reduction of the smoking desire and an improvement in the males' mood. Whereas, in women the same result has been obtained with the e-cigarette and the placebo conditions. Moreover, to explore the e-cigarette impact on cognitive performance, neuropsychological assessment has then been conducted. Results have shown that in the nicotine group, the working memory performance has improved and some differences have emerged with respect to placebo and to just hold groups at a longer interference periods. Our study, partially, is in line with Dawkins results: all cognitive measures attention, executive function and working memory are not influenced by the different e-cigarette and gender showing that in general electronics cigarettes could become a strong support also from a cognitive point of view for those who decide to quit smoking. Differently from Dawkins' study, the interesting result of our research is that there is no difference among electronic cigarettes with or without nicotine; this demonstrates that the daily cognitive performance is not only linked to the presence or to the absence of nicotine. Current smokers who will switch to the electronic cigarette, with or without nicotine, should not fear a reduction in cognitive performance.

For all models of electronic cigarettes breath CO levels did not rise after either series of puffs, thereby confirming that the heating of the solution did not result in combustion. The fact that electronic cigarettes generate heat to aerosolize nicotine, eliminates many of the toxic constituents (e.g., tar and carbon monoxide) created by the combustion of tobacco in classic cigarettes.

We observed a significantly increase of participants' Craving measure in group “C,” “H” “E” and in N and O group condition, we don't have a Craving effect differences. It's likely that after a night of abstinence the re-circulation of significant levels of nicotine dependence reactivate the circuit for which the craving increases progressively in relation to levels of nicotinic placed back in circulation. In fact this phenomenon occurs only for the classic cigarette and for second generation electronic cigarettes. Users of some of the early-generation ENDS products achieved nicotine levels similar to those reached with placebo (Bullen et al., 2010). In contrast, users of the second-generation ENDS products with higher-voltage batteries achieved nicotine levels similar to those reached by smoking classic cigarette (Vansickel and Eissenberg, 2013).

Probably the activation and persistence of craving appears dose depending because with the classic cigarette, craving increases significantly for more time compared whit the two type e-cigarettes. Specifically In C group, craving measure increase over the course of the experiment from T1 to T6 time ratings; also, in H group, craving measure increase over the course of the experiment from T1 to T6 time ratings. In E group, craving measure increase over the course of the experiment form T1 to T3 time ratings (see Figure 3).

Overall, all the products were similar on a range of subjective ratings of user of “smoking gesture,” indicating that for some participants, satisfaction from e-cigarette use was good enough to compensate for their need of own brand cigarette. Indeed the replacement of the ritual of smoking gestures and cigarette handling. E-cig may provide a coping mechanism for conditioned smoking cues by replacing some of the rituals associated with smoking gestures. For example, smoking gestures (e.g., the tactile sensations of the cigarette and other sensations associated with smoking gestures) can play an important part in tobacco addiction as they are usually performed in a predictable, ritualistic manner that act to signal a mental context shift. When the smoker stops smoking the need for the ritual still exists and this is an important cause of relapse. Smoking cessation drugs cannot replace the rituals associated with the act of smoking. Ecig for smoking cessation or smoking reduction is intended to help smokers in coping with this important aspect of their life by implementing personalized replacement rituals.

The strengths of this study include the study addicted of dependent but healthy male and female smokers recruited from the community; the use of a crossover design to minimize variability, bias and confounding.

The study has a number of limitations. First, the small sample of primarily Caucasian, limits the study's generalizability. Second, low baseline ratings of desire to smoke may have limited the degree of observable change. Limitations of the present research include the fact that the exclusion criterion may have imposed restrictions on the sociodemographic variability within our sample; specifically, only individuals smoke ≥15 factory made cigarettes were included in this study. Secondly, a growing prevalence of individuals who smoke are characterized as light—or light and intermittent—smokers it is unknown whether the findings of the current study could be extended to individuals who meet this criteria.

## Conclusion

This is the first study to explore the impact of different e-cigarette models on cognitive performance (executive functions, sustained attention and vigilance, working memory). Our results suggest that neuropsychological function are not affected by group condition. The group without nicotine, that is using the “one mint” cigarette, shows the same score of the other four groups in the test used for to evaluate attention, executive functions and working memory. Therefore, the nicotine presence does not interfere with the cognitive functioning that is evaluated in its different components. Hence smokers who will switch to the electronic cigarette, with or without nicotine, should not fear a reduction in cognitive performance. It is interesting to note that the group without nicotine is different from the others due to the nicotine aroma, since the menthol cigarettes have been recently analyzed by the Food and Drug administration in USA (FDA). This variant of e-cigarette has been considered responsible to favor the dependence to smoking exactly when the mint aroma would make the breathed smoke less bitter, with a less dangerous effect for the smoker. More recent findings have demonstrated menthol role in the metabolism of nicotine in the body through the nicotinic acetylcholine (nACh) receptor in cells. This receptor is essential to the actions of nicotine in the brain showing an important role in nicotine addiction (Kabbani, 2013). It seems that not only craving and other smoke withdrawal symptoms but also cognitive performance is not only linked to the presence of nicotine; this suggests that the reasons behind the dependence and the related difficulty to quit smoking needs to be looked into also other factors like smokers identity, smoking rituals and the gesture.

## Ethics statement

The study was approved by University of Catania Institutional Ethics Committee.

## Author contributions

All authors listed, have made substantial, direct and intellectual contribution to the work, and approved it for publication.

### Conflict of interest statement

The authors declare that the research was conducted in the absence of any commercial or financial relationships that could be construed as a potential conflict of interest.

## Footnotes

^1^World Health Organization (WHO) report on the global tobacco epidemic, 2008 www.who.int/tobacco/mpower/2008/en/index.html.

^2^The health benefits of smoking cessation In. Edited by Services UDoHaH; USA, US Department of Health and Human Services, Public Health Service, Centers for Disease Control, Center for Chronic Disease Prevention and Health Promotion, Office on Smoking and Health; 1990.

^3^World Health Organization (WHO), WHO Framework Convention on Tobacco Control. ISBN: 9241591013.
